# Re-challenging pralsetinib following recovery from pneumocystis jirovecii pneumonia in a lung cancer patient: a Case Report

**DOI:** 10.3389/fphar.2024.1443609

**Published:** 2025-02-27

**Authors:** Zhe Zhao, Longbin Pang, Surui Liu, Jie Liu

**Affiliations:** ^1^ Department of Oncology, Jinan Central Hospital, Shandong University, Jinan, Shandong, China; ^2^ Pulmonary and Critical Care Medicine, Central Hospital Affiliated to Shandong First Medical University, Jinan, Shandong, China; ^3^ Department of Oncology, Central Hospital Affiliated to Shandong First Medical University, Jinan, Shandong, China

**Keywords:** pralsetinib, RET fusion, pneumocystis jirovecii pneumonia, non-small cell lung cancer, case report

## Abstract

**Background:**

Pneumocystis jirovecii pneumonia (PJP), an opportunistic infection, is commonly observed in immunocompromised individuals, particularly those with cancer, and is known for its significant morbidity and mortality rates. Pralsetinib is a highly specific inhibitor that targets advanced or metastatic non-small cell lung cancer (NSCLC) characterized by RET-fusion positivity. The incidence of PJP infection in patients receiving pralsetinib was found to be infrequent. However, there is currently a lack of consensus regarding the rechallenge of pralsetinib in patients who have fully recovered from PJP.

**Case presentation:**

In this case study, a 60-year-old patient diagnosed with stage IV lung adenocarcinoma and carrying a KIF5B-RET fusion gene underwent pralsetinib treatment as the fourth-line therapy. Subsequently, the patient developed a fever and dyspnea 2.5 months later. However, the patient did not exhibit a positive response to the empirical antibiotic therapy administered. The computed tomography findings indicated widespread ground-glass opacities with numerous cystic lesions in both lungs, along with patchy consolidations in the lower right lung. The diagnosis of PJP was conclusively confirmed through bronchoalveolar lavage. The patient’s condition was effectively treated with a combination of oral trimethoprim/sulfamethoxazole and intravenous caspofungin along with clindamycin. The patient fully recovered from PJP. Subsequently, he underwent a rechallenge with pralsetinib, and as of the latest follow-up, no evidence of progressive disease has been observed.

**Conclusion:**

This case report emphasizes the significance for physicians to be cognizant of the potential hazard of PJP development in cancer patients undergoing pralsetinib treatment, particularly in those who are unresponsive to empirical antibiotic therapy. Prompt identification and timely intervention are essential to achieve better outcomes in patients with pralsetinib-induced PJP. Furthermore, it highlights the scenario where patients who have fully recovered from moderate-to-severe pralsetinib-induced PJP may undergo pralsetinib re-administration without requiring alternative treatment options.

## Introduction

RET fusions and mutations serve as oncogenic drivers of a variety of tumors, and are found in 1%–2% of non-small cell lung cancers (NSCLC) (Gainor and Shaw, 2013). The standard treatment for these patients is targeted chemotherapy agents. Multikinase inhibitors, such as cabozantinib, lenvatinib, and vandetanib, have been explored and have shown low response rates (16%–47%) in clinical investigations (Lee et al., 2017; Yoh et al., 2017; Hida et al., 2019; Drilon et al., 2016). Pralsetinib (BLU-667) is an oral, potent, and highly selective kinase inhibitor targeting RET variants. ARROW is the first prospective study to investigate pralsetinib for the treatment of RET fusion-positive NSCLC and in an updated analysis from the trial, ORR was 62% (95% CI 53–70) in patients who had received prior platinum therapy (n = 126) and 73% (95% CI 50–89) in those who had received prior non-platinum therapy (n = 22); The median PFS was 16.5 and 12.8 months, respectively (Griesinger et al., 2022). Based on these findings, the FDA in the U.S. granted approval for the treatment of advanced or metastatic RET fusion-positive NSCLC.

The most prevalent grade 3 or higher treatment-related adverse events included neutropenia [43 patients (18%)], hypertension [26 (11%)], and anemia [24 (10%)] in the ARROW group (Griesinger et al., 2022). Pneumonitis, graded three or higher, was reported in 2% of patients with RET fusion-positive NSCLC (Gainor et al., 2021). Pneumocystis jirovecii pneumonia (PJP), an opportunistic fungal infection, is frequently observed in immunocompromised patients, encompassing those with AIDS, cancer, and organ transplants (Thomas and Limper, 2004), with mortality rates exceeding 20% (Morris et al., 2004). However, there are limited case reports documenting pralsetinib-induced PJP. The role of pralsetinib rechallenge remains undefined in cancer patients who have discontinued treatment due to PJP-associated toxicity. In this context, we present a case of NSCLC that developed grade 3 pneumonitis following pralsetinib as fourth-line therapy. The patient exhibited an improved response to the pralsetinib rechallenge, demonstrating good safety after complete recovery from PJP.

## Case report

A 60-year-old male patient received a diagnosis of lung adenocarcinoma in April 2019 and underwent a right upper lobectomy along with visible pleural nodule resection on 26 April 2019. The postoperative pathological examination confirmed the presence of adenocarcinoma with malignant pleural dissemination. According to the eighth edition American Joint Committee on Cancer (AJCC) staging system, the postoperative staging was determined as pT2aN1M1a, stage IVA. The KIF5B-RET fusion was identified through next-generation sequencing (NGS) on 9 May 2019. The patient underwent a series of treatments. The first-line therapy included pemetrexed, cisplatin, and pembrolizumab. Subsequently, second-line therapy comprised paclitaxel, carboplatin, and bevacizumab. Finally, anlotinib was administered as third-line therapy. However, these treatments were discontinued due to either tumor progression or intolerance. Following this, the patient commenced pralsetinib as fourth-line treatment, administered orally at a daily dose of 400 mg starting from 10 February 2022.

**FIGURE 1:**
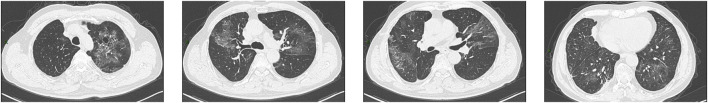
CT images obtained on 5 May 2022. The images showed diffuse ground-glass opacities with numerous cystic lesions in both lungs and patchy consolidations in the right lower lung.

**FIGURE 2:**
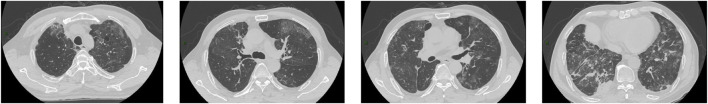
CT images obtained on 11 May 2022. The images showed the range of the lesions further extended with the interlobular septal thickening.

Throughout treatment, the patient encountered grade 2 neutropenia according to the National Cancer Institute Common Toxicity Criteria for Adverse Events (NCI-CTCAE, version 5.0). On 3 May 2022, the patient was hospitalized due to a fever of 38.6°C. With an Eastern Cooperative Oncology Group (ECOG) performance status of 1, the patient maintained hemodynamic stability. Oxygen saturation levels were measured at 97% without the need for supplemental oxygen. Auscultation revealed coarse breath sounds bilaterally, accompanied by a few dry rales. Subsequent routine blood tests conducted on the same day disclosed a decline in lymphocyte count (absolute lymphocyte value of 0.54×10^9^/L, reference range 1.1–3.2×10^9^/L) and an elevation in C-reactive protein (CRP) levels (CRP 218.58 mg/L, reference range 0–3.0 mg/L). Following an assessment indicating community-acquired pneumonia, we initiated the intravenous infusion of levofloxacin (0.4 g/24 h). However, 48 h post-admission, the patient developed severe dyspnea, leading to a decrease in oxygen saturation to 90% (without supplemental oxygen), necessitating the use of 40% oxygen to maintain saturation above the established normal threshold (>95%). On 5 May 2022, a computed tomography (CT) scan revealed widespread ground-glass opacities with numerous cystic lesions bilaterally, alongside localized consolidations in the lower right lung (Figure 1). The patient exhibited persistent fever (37.4°C) after admission. Consequently, pralsetinib was halted, and the therapeutic regimen was modified to incorporate moxifloxacin (0.4 g/24 h) along with oseltamivir (75 mg twice daily) in an empirical approach. Furthermore, sputum culture and a battery of tests for respiratory pathogens, cytomegalovirus, and herpes simplex virus DNA assays were conducted. Unfortunately, none of these tests provided informative results. Subsequently, bronchoalveolar lavage (BAL) fluid samples were obtained from the lungs via fiberoptic bronchoscopy for NGS. Despite these interventions, the patient’s clinical condition deteriorated, and oxygen saturation remained at 50%. A subsequent CT scan revealed an expanded range of lesions characterized by interlobular septal thickening (Figure 2). Considering the CT findings and the patient’s clinical presentation, pneumocystis pneumonia was highly probable, prompting the administration of trimethoprim/sulfamethoxazole (TMP-SMZ, 160/800 mg, every 12 h), caspofungin (50 mg/24 h), and clindamycin for anti-infective treatment. Subsequent BAL results confirmed our clinical suspicion, demonstrating positivity for P. jirovecii with a sequence number of 61,636. Consequently, the patient’s condition improved, as reflected in enhanced pulmonary auscultation findings, resolution of fever, and improved blood test results. Due to the favorable progression of his condition, the patient expressed a desire for early discharge after receiving treatment with TMP-SMZ (160/800 mg every 8 h), caspofungin (50 mg/24 h), and clindamycin (0.6 g/8 h) for 3 days. Subsequently, the patient was administered TMP-SMZ (every alternate day) for 2 weeks. The severity of PJP in this patient was classified as grade 3 according to CTCAE.

**FIGURE 3:**
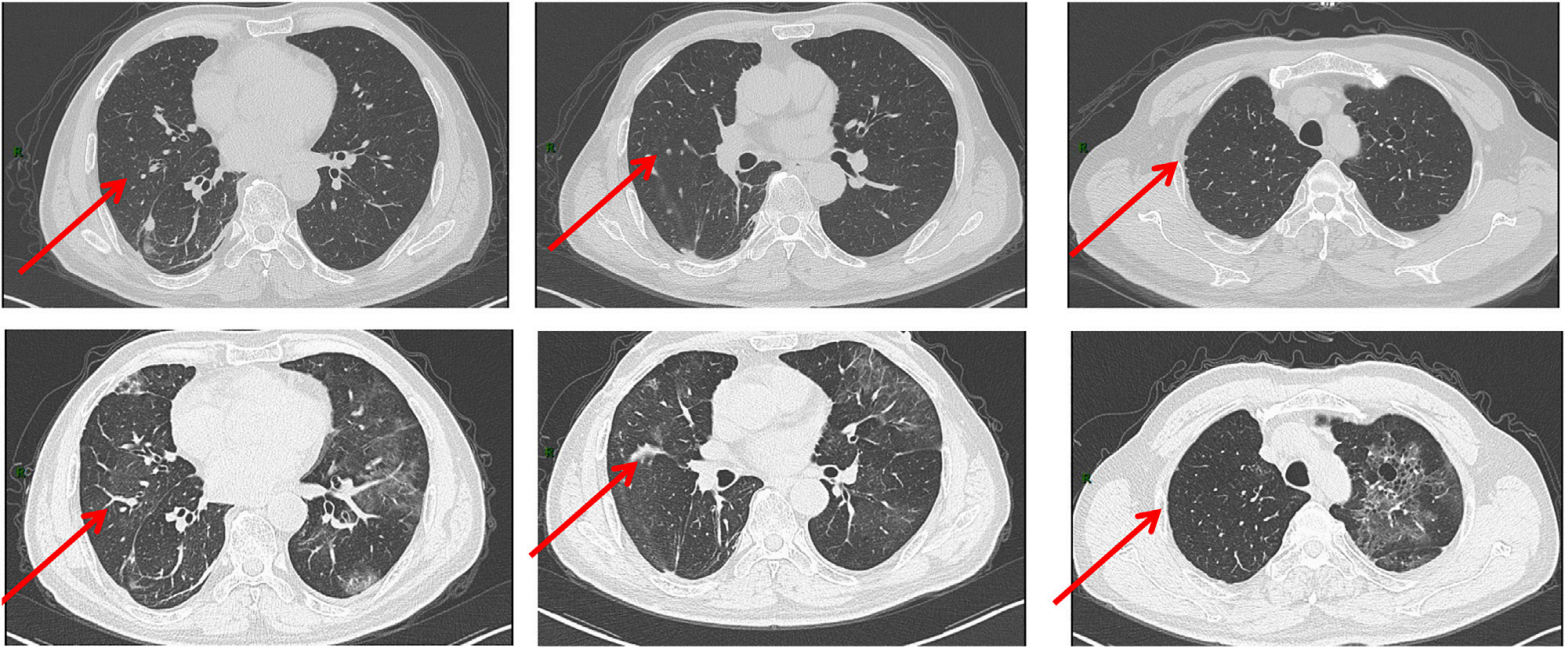
CT images obtained on 2 March 2022(UP) and 5 May 2022 (DOWN). The size of the metastatic lesion was reduced after pralsetinib treatment.

**FIGURE 4:**
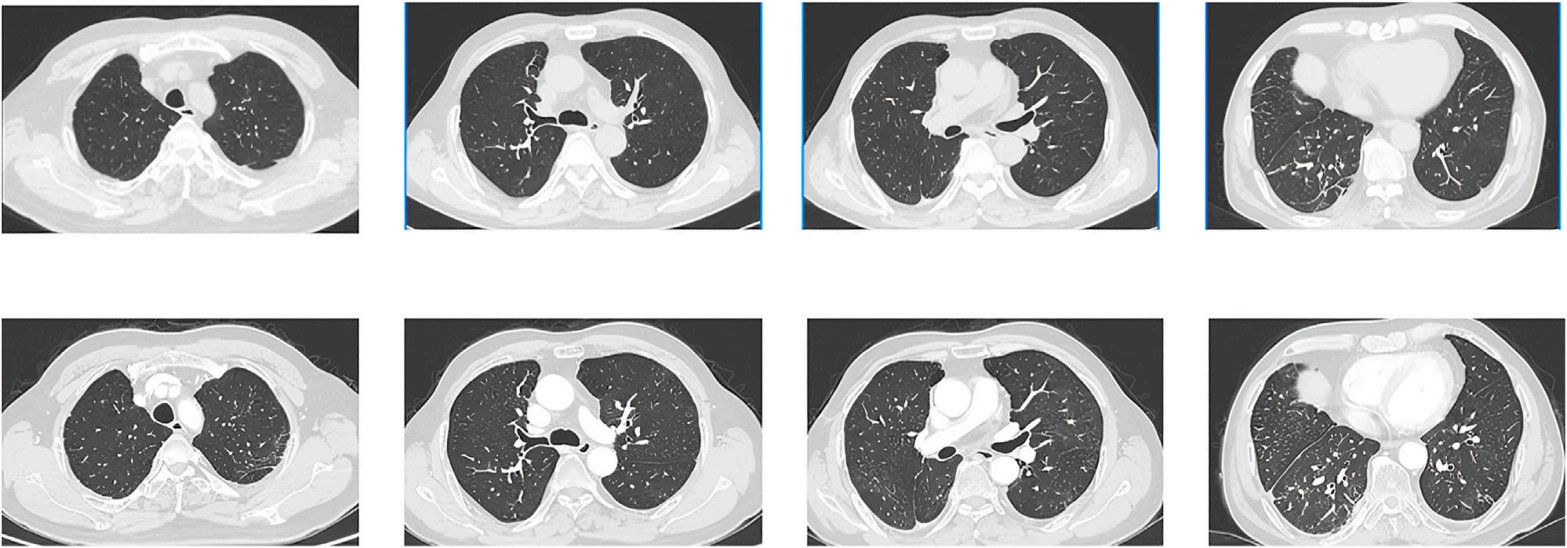
CT images obtained on 2 September 2022(UP) and 10 October 2023 (DOWN). The images showed that the ground-glass opacities, cystic lesions, and patchy consolidations had completely disappeared, and metastatic lesion size continued to be reduced.

It is noteworthy that the CT conducted on May 5th revealed a reduction in the size of the metastatic lesion (Figure 3), and the efficacy assessment indicated a partial response (PR) based on the Response Evaluation Criteria in Solid Tumors version 1.1 (RECIST 1.1). Due to the limited availability of alternative therapeutic approaches, patients have continued their antitumor therapy with pralsetinib. Subsequently, a follow-up visit was conducted after 3 months, during which the CT scan demonstrated the complete disappearance of ground-glass opacities, cystic lesions, and patchy consolidations, along with a further decrease in the size of the metastatic lesion (Figure 4). Over the ensuing 13 months, the patient faithfully adhered to the prescribed regimen of Pralatinib, and was regularly rechecked in the hospital, with no significant change in tumor size noted and no recurrence of any adverse events (Figure 4).

## Discussion

Pneumocystis jirovecii pneumonia (PJP) is typically seen in immunocompromised individuals like those with HIV/AIDS. However, recent studies note an increasing occurrence in HIV-negative patients, particularly those with hematological malignancies and solid tumors. Factors such as corticosteroid use, lymphocytopenia, chest radiation, and recent chemotherapy may contribute to PJP development (Takeda et al., 2021). A separate investigation yielded comparable findings, suggesting that patients undergoing treatment for lung cancer with radical radiotherapy may face an increased susceptibility to PJP (McAleese et al., 2018). It has been proposed that the occurrence of PJP is influenced by the disease stage and treatment protocols (Takeuchi and Yakushijin, 2021). Certain cancer treatments, like chemotherapy for lung cancer and drugs targeting specific molecular pathways, have been associated with increased PJP risk (Doello et al., 2020; Khoo et al., 2019; Watanabe et al., 2020). For instance, olaparib, a poly ADP-ribose polymerase (PARP) inhibitor (PARPi), has been linked to PJP in an analysis of the Food and Drug Administration Adverse Event Reporting System (FAERS) (Shu et al., 2022). Checkpoint inhibitors, especially when steroid use is necessary (Shah et al., 2022), and drugs like pralsetinib may also elevate PJP susceptibility, possibly due to immune suppression (Lee et al., 2021). Further research is needed to understand these connections and implications for drug development.

PJP typically presents with fever, chest tightness, difficulty breathing, and respiratory failure. Chest CT scans show characteristic symmetrical ground-glass opacities, mainly in the upper lobes. Due to the nonspecific symptoms and imaging findings, a thorough differential diagnosis is crucial. In patients treated with pralsetinib, distinguishing between drug-induced pneumonitis and PJP can be challenging. Clinicians should consult a multidisciplinary team (MDT)early if antibiotic treatment is ineffective. Diagnosis often involves bronchoscopy and bronchoalveolar lavage, with culture as a benchmark. Next-generation sequencing aids in detecting atypical pathogens, confirming PJP.

Studies suggest promptly discontinuing pralsetinib if patients experience severe pulmonary complications like pneumonia or respiratory distress. Guidelines recommend permanent discontinuation for grade 3 or more severe pralsetinib-induced pneumonitis, but it is unclear if grade ≥3 PJP warrants the same. A patient in this case report recovered fully from PJP with treatment and showed no signs of pneumonia upon discharge. Despite limited alternative options, the patient consented to continue pralsetinib with intensified monitoring, experiencing no further issues. While this suggests favorable outcomes with pralsetinib, conclusions are based on limited case studies, urging the need for larger multi-center trials to validate findings in rare mutation patients.

## Conclusion

Pneumocystis jirovecii pneumonia (PJP) is infrequent among individuals with cancer; nevertheless, it is crucial to acknowledge the potential hazard of PJP development in cancer patients undergoing pralsetinib therapy, especially in those unresponsive to empirical antibiotic treatment. Prompt identification and timely intervention play a pivotal role in enhancing patient outcomes in cases of pralsetinib-induced PJP. Additionally, this underscores the possibility of reintroducing pralsetinib as a treatment option without necessitating the selection of an alternative therapy following complete recovery from moderate-to-severe pralsetinib-induced PJP.

## Data Availability

The original contributions presented in the study are included in the article; further inquiries can be directed to the corresponding author.

## Abbreviations

NSCLC, non-small cell lung cancer; PJP, Pneumocystis jirovecii pneumonia; AJCC, American Joint Committee on Cancer; NGS, next-generation sequencing; NCI-CTCAE, National Cancer Institute Common Toxicity Criteria for Adverse Events; ECOG, Eastern Cooperative Oncology Group; CRP, C-reactive protein; CT, computed tomography; BAL, bronchoalveolar lavage; TMP-SMZ, trimethoprim/sulfamethoxazole; PR, partial response; RECIST, Response Evaluation Criteria in Solid Tumors; TRAEs, treatment-related adverse events; PARP, poly ADP-ribose polymerase; PARPi, poly ADP-ribose polymerase inhibitor; FAERS, Food and Drug Administration Adverse Event Reporting System; MDT, multidisciplinary team.
